# Health of Philadelphia

**Published:** 1845-11

**Authors:** 


### THE MEDICAL EXAMINER.

### PHILADELPHIA, NOV., 1845.

### HEALTH OF PHILADELPHIA.

The Reports of the Board of Health, as well as the observations of the most extensive practitioners of medicine, show the present to be an extraordinarily healthy season in Philadelphia. We hear of small-pox in some of the neighbouring cities, but in this place we know of no contagious, epidemic, or other diseases of a serious character—nothing more than the ordinary occurrences, such as colds, accidents, &c. The season has been mild, and the autumn, thus far, more exempt from storms and foul weather than we have almost ever experienced.

